# The Cumulative Effects of Predictability on Synaptic Gain in the Auditory Processing Stream

**DOI:** 10.1523/JNEUROSCI.0291-17.2017

**Published:** 2017-07-12

**Authors:** Ryszard Auksztulewicz, Nicolas Barascud, Gerald Cooray, Anna Christina Nobre, Maria Chait, Karl Friston

**Affiliations:** ^1^Oxford Centre for Human Brain Activity, University of Oxford, Oxford OX3 7JX, United Kingdom,; ^2^Wellcome Trust Centre for Neuroimaging, UCL, London WC1N 3BG, United Kingdom,; ^3^Ear Institute, University College London, London WC1X 8EE, United Kingdom,; ^4^LSCP, Département d'Etudes Cognitives, ENS, EHESS, CNRS, PSL Research University, 75005 Paris, France, and; ^5^Clinical Neuroscience, Karolinska University Hospital, 171 76 Stockholm, Sweden

**Keywords:** auditory processing, dynamic causal modeling, gain modulation, magnetoencephalography, neural oscillations, predictive coding

## Abstract

Stimulus predictability can lead to substantial modulations of brain activity, such as shifts in sustained magnetic field amplitude, measured with magnetoencephalography (MEG). Here, we provide a mechanistic explanation of these effects using MEG data acquired from healthy human volunteers (*N* = 13, 7 female). In a source-level analysis of induced responses, we established the effects of orthogonal predictability manipulations of rapid tone-pip sequences (namely, sequence regularity and alphabet size) along the auditory processing stream. In auditory cortex, regular sequences with smaller alphabets induced greater gamma activity. Furthermore, sequence regularity shifted induced activity in frontal regions toward higher frequencies. To model these effects in terms of the underlying neurophysiology, we used dynamic causal modeling for cross-spectral density and estimated slow fluctuations in neural (postsynaptic) gain. Using the model-based parameters, we accurately explain the sensor-level sustained field amplitude, demonstrating that slow changes in synaptic efficacy, combined with sustained sensory input, can result in profound and sustained effects on neural responses to predictable sensory streams.

**SIGNIFICANCE STATEMENT** Brain activity can be strongly modulated by the predictability of stimuli it is currently processing. An example of such a modulation is a shift in sustained magnetic field amplitude, measured with magnetoencephalography. Here, we provide a mechanistic explanation of these effects. First, we establish the oscillatory neural correlates of independent predictability manipulations in hierarchically distinct areas of the auditory processing stream. Next, we use a biophysically realistic computational model to explain these effects in terms of the underlying neurophysiology. Finally, using the model-based parameters describing neural gain modulation, we can explain the previously unexplained effects observed at the sensor level. This demonstrates that slow modulations of synaptic gain can result in profound and sustained effects on neural activity.

## Introduction

The faculty to recognize or learn sensory regularities has been shown in many domains, from sensitivity to stimulus statistics in anesthetized animals (Yaron et al., 2012) to complex decision-making behavior (for review, see Summerfield and de Lange, 2014). Converging evidence suggests that stimulus predictability shapes processing at the level of sensory cortex by modulating postsynaptic efficacy or gain (Moran et al., 2013; for review, see Schröger et al., 2015). Even within the same region, neurons can be sensitive to sensory regularities at different time scales, as shown for the auditory cortex in the context of stimulus-specific adaptation (Ulanovsky et al., 2004; Rubin et al., 2016). Recent work suggests that the modulatory effects of predictability may extend to subcortical regions (Ohmae et al., 2013; Pérez-González and Malmierca, 2014) and higher cortical areas (Rao et al., 2012; Cheadle et al., 2014). How different levels of the cortical hierarchy orchestrate the modulation of neuronal activity is an outstanding and important question.

Explaining predictability in terms of postsynaptic gain control is intuitively accommodated by generalized predictive coding (Friston, 2005), where stimulus predictability is directly linked to the precision of sensory signals in the brain. Under predictive coding, the brain is assumed to continuously generate descending inhibitory signals, conveying predictions about sensory inputs, that attenuate signals ascending from the superficial layers of sensory regions. However, predictability can also increase synaptic gain or efficacy in superficial layers (Kanai et al., 2015), reflecting increased confidence in, or attention to, ascending prediction errors (Feldman and Friston, 2010). Crucially, this can reverse the attenuation of ascending signals by descending predictions (Kok et al., 2012). Synaptic gain control, by modulating the excitability of principal cells and neuronal time constants (Bastos et al., 2012) can thus shift-induced activity from lower to higher frequencies (Chawla et al., 1999); a phenomenon known as *synchronous gain*. This is equivalent to increasing the power of gamma-band oscillations and decreasing the power of alpha oscillations, a commonly reported correlate of predictability (Arnal et al., 2011; Todorovic et al., 2011; Bauer et al., 2014; Brodski et al., 2015; Sedley et al., 2016).

In addition to effects on synchronous activity, a recent study (Barascud et al., 2016) has shown that adding a repetitive structure to an auditory stimulus sequence resulted in a substantial shift in the sustained field amplitude, as measured using magnetoencephalography (MEG). In a passive listening task, they used rapid tone-pip sequences comprising a regularly repeating frequency pattern (REG), as well as frequency-matched sequences of tones presented in a random order (RAND), while systematically varying the number of unique frequencies in each sequence (alphabet size). Regular sequences were associated with higher MEG sustained response amplitudes, relative to their random counterparts. In addition, RAND sequences associated with smaller alphabet sizes elicited stronger sustained responses, such that the amplitude modulation appeared to correlate with the predictability of the sequences. This effect has also been recently replicated using electroencephalography (Southwell et al., 2017). Sustained MEG responses were previously found to colocalize with broadband gamma synchronization and alpha desynchronization (Brookes et al., 2005). However, context-sensitive slow modulations of local field potentials in the macaque visual cortex were reported to be uncorrelated with broadband gamma power (Ray and Maunsell, 2011), raising the possibility that sustained fields are underpinned by network dynamics only indirectly related to spiking activity.

Here, we tested whether stimulus predictability has an effect on high-frequency activity that could be explained by sustained modulation of postsynaptic gain. Having established the effects of predictability on induced activity in different sources along the auditory pathway, we used biophysically realistic (dynamic causal) modeling of spectral responses to explain the modulations of induced responses in terms of slowly fluctuating changes in (intrinsic) synaptic efficacy within regions and (extrinsic) synaptic connectivity between sources. Finally, we used estimates of condition-specific changes in (intrinsic and extrinsic) synaptic efficacy to see whether they could account for the condition-specific changes in sustained field amplitude.

## Materials and Methods

### 

#### 

##### Experimental design and statistical analyses.

The present results are based on a reanalysis of the data originally reported by Barascud et al. (2016, Experiment 4). Thirteen healthy participants (6 male; mean age, 28 ± 8 years) with no history of hearing or neurological disorders were enrolled in the study upon written informed consent. All experimental procedures were approved by the research ethics committee at University College London. Participants had no prior exposure to the auditory sequences used in this study and were engaged by an incidental visual task (N-back, consisting of a sequence of landscape images). The experiment was divided into four runs of ∼10 min. The original paradigm and an analysis of MEG sustained fields were reported in detail by Barascud et al. (2016, Experiment 4).

**Figure 1.**
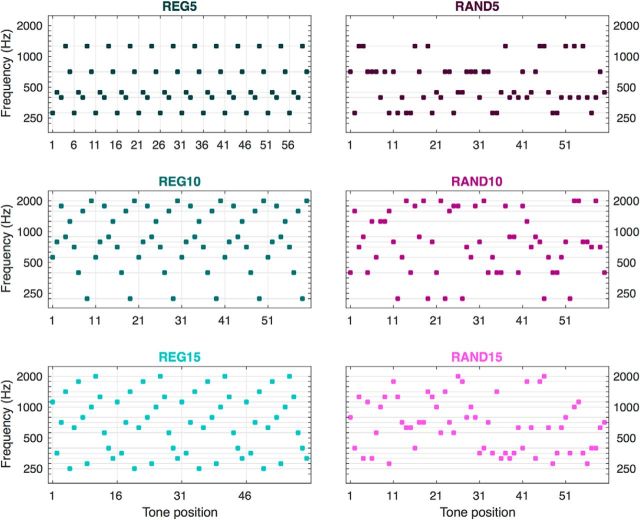
Schematic example of the sound stimuli. Each dot symbolizes a 50 ms tone pip. Left, REG signals. Sequences were generated by selecting (randomly, with replacement) 5, 10, or 15 tones (“alphabet”) from the frequency pool and repeating that order. Horizontal gray lines show the alphabet (i.e., the available frequencies for a given stimulus) for the example sequence, vertical gray lines indicate the first tone of each cycle. Right, Matched RAND signals were generated by sampling randomly from the same alphabet.

Auditory stimuli are illustrated in Figure 1. They were sequences of abutting tone-pips (50 ms duration; gated at onset and offset with 5 ms raised cosine ramps) lasting 3000 ms. Individual tone-pip frequencies were drawn from a range between 222 and 2000 Hz in 20 equally spaced steps on a logarithmic scale. The auditory sequences were subject to two experimental manipulations: (1) *regularity*, with auditory sequences consisting either of regularly repeated blocks of several frequencies selected (randomly, with replacement) from the frequency pool (REG condition), or of a matched subset of frequencies presented in a random order (RAND condition); (2) *alphabet size*, determining the number *R* of frequencies in the repeated REG cycle or in the alphabet used to generate the RAND sequence (*r* = 5, 10, or 15 frequencies). The original stimulus set also included RAND20 sequences, sampling the entire frequency pool (alphabet size of 20), which were left out of the present analysis to ensure a balanced design. Specifically, in subsequent modeling (see Dynamic causal modeling), we wanted to preclude unmatched experimental conditions from confounding our modeling of the differences between the conditions.

Per participant and condition, 104 different sequences were generated. The order of sequence presentation was randomized, such that the sequence type was unpredictable on a given trial. Sequences were separated by a random interval between 700 and 2000 ms. Stimulus presentation was controlled via the Cogent toolbox (http://www.vislab.ucl.ac.uk/cogent.php) for MATLAB (MathWorks). Sounds were rendered offline, stored as 16 bit WAV files at 44.1 kHz, and delivered using (EARTONE 3A 10 Ω, Etymotic Research) headphones at a comfortable listening level (self-adjusted by each listener).

Details of statistical analyses are reported separately for sensor-level data (below), time-frequency data (see Time-frequency analysis), and dynamic causal modeling (see Dynamic causal modeling).

##### MEG data acquisition and sensor-level analysis.

MEG signals were acquired in a 275-channel whole-head setup with third-order gradiometers (CTF Systems) at a sampling rate of 600 Hz. All analyses were performed using a MATLAB (MathWorks) toolbox SPM12 (Wellcome Trust Centre for Neuroimaging, University College London). Continuous data were high-pass filtered at 0.1 Hz, notch-filtered at 50 Hz and harmonics, and epoched into segments time-locked to stimulation window onset (from −700 to 3700 ms peristimulus time). Epochs with *z*-score amplitudes and amplitude differences >6 SD of all trials were automatically rejected.

**Figure 2.**
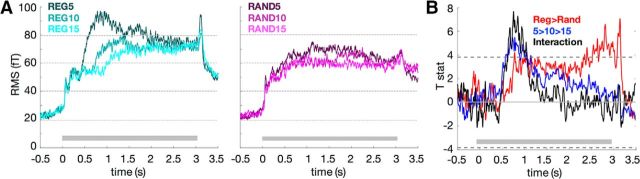
***A***, Sensor-level root mean square (RMS) signals (over all MEG channels) evoked by auditory sequences of varying alphabet size (5, 10, or 15 tones) and predictability (left, regular/repeating sequences; right, random sequences; cf. Barascud et al., 2016). Gray bars show auditory sequence duration. ***B***, Sensor-level effects of predictability (regular > random sequences), alphabet size, and their interaction. Dashed lines mark threshold of significance while correcting for multiple comparisons at a FWE rate 0.05.

The sensor-level results were previously analyzed and reported by Barascud et al. (2016). Here, we reanalyzed the data with slightly different preprocessing settings (i.e., without PCA-based bias on precisely evoked activity, and using high-pass filtering at 0.1 Hz which preserves quasi-stationarity of the signals, necessary for subsequent dynamic causal modeling of cross-spectral density) and focused on the main and interaction effects of regularity and alphabet size on the amplitude of the sustained field (Fig. 2).

##### Source reconstruction.

Sensor-level data were source-localized using a multiple-sparse-priors (greedy search) approach (Friston et al., 2008). Following averaging across conditions and participants, and an inversion (source localization or fitting) of the grand-averaged sensor-level data over the whole time window (from −700 to 3700 ms peristimulus time), estimated source activity maps were converted into 3D images for an early time-window (0–500 ms poststimulus onset) and the corresponding baseline (−500–0 ms prestimulus). The early time window, in which responses to all signals are overlapping, was chosen for source localization to ensure that condition-specific differences did not bias the selection of sources, but instead reflect generic processing of sequences. Evoked power was calculated by subtracting the baseline from the poststimulus activity map. Sources of the largest evoked activity were identified as clusters of voxels whose evoked power was larger than the mean + 2 SD of all positive values in the evoked power map. These clusters were then used to extract individual subjects' source-level time-series using a linearly constrained minimum variance beamformer (Van Veen et al., 1997), as implemented in the Data Analysis in Source Space (DAiSS) toolbox for SPM12 (https://github.com/SPM/DAiSS), separately for each condition. Sources were labeled using the SPM12 atlas provided by Neuromorphometrics, and the Talairach Daemon gyrus-level atlas (Lancaster et al., 2000).

##### Time-frequency analysis.

Based on the source-level time-series, time-frequency maps were estimated using wavelet decomposition with a Hanning taper (window length: 500 ms, time-step: 50 ms, frequency range: 8–128 Hz, frequency resolution: 2 Hz). In the analysis of induced responses, single-trial time-frequency estimates (after subtracting the averaged evoked response from each trial) were log-rescaled to a prestimulus baseline (from −450 to −250 ms relative to stimulus onset). The baseline was chosen to preclude contamination by acoustic stimulation on a given trial (up to −250 ms, given window length of 500 ms) or on a previous trial (up to −450 ms, given 700 ms as the shortest interval between 2 sequences). Rescaling was implemented as a difference between the logarithms of poststimulus and baseline time-frequency estimates. Data were averaged across trials using robust averaging (Wager et al., 2005), which iteratively down-weights the influence of trials with outliers on the average. Subject-specific time-frequency maps were converted into 2D images per region and condition and smoothed using a Gaussian kernel (8 Hz and 500 ms full-width at half-maximum) to meet the assumptions of random field theory (Kilner et al., 2005). To test for differences in induced power between hemispheres, the images were entered into 2 × 6 factorial GLMs with within-subject factors Hemisphere (left vs right) and Condition. The *p* values of the statistical parametric maps were corrected for multiple comparisons based upon time-frequency cluster size using a familywise error (FWE) rate at *p* < 0.05. Because no differences in induced power were observed between hemispheres (*F* tests for both regions identified in the source reconstructions: all voxels *p*_FWE_ > 0.05), the resulting rescaled time-frequency maps were averaged across hemispheres for each of the sources identified in the source reconstruction. For subsequent tests of the effects of experimental conditions, images were entered into a 2 × 3 factorial GLM with within-subject factors regularity (random vs regular sequence of tones) and alphabet size (alphabet size of 5, 10, or 15 tones). Because, by design, the regularity of sequences of different length takes a different time to be discovered, significant effects were inferred for both sources identified in the source reconstruction (see Results) within a 750–3000 ms time window, chosen to ensure that sequence regularity can in principle be discovered (thus, after 750 ms for REG15), and differences in induced power can be ascribed to sustained neural processing of predictability rather than mere statistics of stimulation. The *p* values of the statistical parametric maps were corrected for multiple comparisons based upon time-frequency cluster size using a FWE rate at *p* < 0.05. To test for the stationarity components of time-frequency effects, we implemented augmented Dickey–Fuller tests, allowing us to infer the presence of a unit root in a time series. This procedure revealed no significant departures from stationarity (under a 250 ms lag order of the autoregressive process), when correcting for multiple comparisons at a liberal false discovery ratio of 0.5. Thus, an identical analysis was performed on frequency spectra averaged across time points over the duration of the stimulus sequence (750–3000 ms).

In a control analysis, aiming to establish whether the significant time-frequency regions can be indeed attributed to the sources identified in the preceding source reconstruction step, we used dynamic imaging of sources (Gross et al., 2001), as implemented in the DAiSS toolbox. This allowed testing for interactions throughout source space. High-frequency (76–86 Hz) power was estimated for the 1250–1750 ms time window, separately for each experimental condition. The resulting whole-brain power images were entered into a general linear model and tested for the interaction effect (REG5 > REG15 × RAND5 < RAND15) identified in the time-frequency analysis. The statistical parametric maps were thresholded at *p* < 0.001 (peak-level, uncorrected) and *p* values were corrected for multiple comparisons based upon 3D volumetric cluster size, using a FWE rate at *p* < 0.05. Sources were labeled using the SPM12 atlas provided by Neuromorphometrics.

##### Dynamic causal modeling.

To model the effects of stimulus regularity and alphabet size on spectral responses in terms of the underlying neurophysiology, data were modeled using DCM for cross-spectral density (Friston et al., 2012). DCM explains observed neural responses using biologically realistic mean-field models of coupled dynamical systems. Neurophysiological inference using DCM has been validated in animal models (Moran et al., 2011) and invasive recordings in humans (Papadopoulou et al., 2015). Crucially, convergent DCM-based inference has been demonstrated using MEG and invasive electrocorticography data (Phillips et al., 2016). Because DCM parameters typically have a physiological interpretation, DCM can be validated using multiple modalities; in particular, using invasive and noninvasive electrophysiological data. Here, the grand-averaged spectra were modeled in a network of coupled cortical sources (based on the significant time-frequency effects described above). Each cortical source comprised four neural populations: pyramidal cells in supragranular and infragranular layers, spiny stellate cells, and inhibitory interneurons (see Fig. 4). This canonical microcircuit model (Pinotsis et al., 2013) was chosen because it offers a relatively straightforward mapping between the DCM parameters and the predictive coding framework (Bastos et al., 2012) and has been used in several other DCM studies of cross-spectral densities (Pinotsis et al., 2014; Bastos et al., 2015; Cooray et al., 2015; Papadopoulou et al., 2015).

The dynamics at each source can be described by the following coupled differential equations:

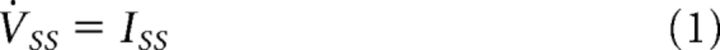


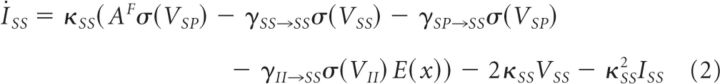


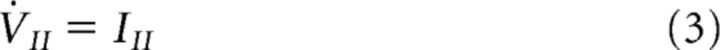


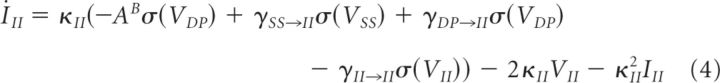


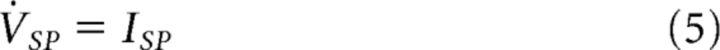


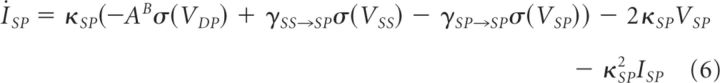


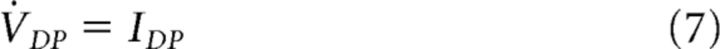


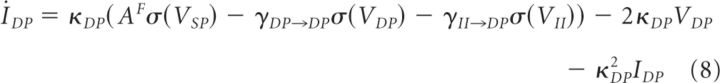
 Here, subscripts denote neuronal populations (SS, spiny stellate cells; II, inhibitory interneurons; SP, superficial pyramidal cells; DP, deep pyramidal cells). The voltage and current of each population *m* are denoted by *V*_m_ *a*nd *I*_m_ respectively, with a synaptic rate constant κ_m_. The postsynaptic potential is transformed into the firing rate by a sigmoid operator σ. *A^F^* and *A^B^* denote the forward and backward (extrinsic) connections between regions and γ_*m*→*n*_ denote the (intrinsic) connection from population *m* to *n* within a region. Finally, spiny stellate cells receive endogenous input *E*(*x*). In DCM for cross-spectral density, this endogenous input is modeled as a linear mixture of firing rates σ(*V*_m_) in all populations.

In the first step, to provide a good fit of the models to the data, prior rate constants κ_m_ and intrinsic connectivity parameters γ_m→*n*_ of the canonical microcircuit model were optimized to reproduce the spectral peaks observed in our dataset, averaged across both regions and experimental conditions. After this initial optimization (see Table 3, prior means), a two-source model (including sources in the putative auditory cortex and the inferior frontal gyrus, identified bilaterally in source localization (see Results, Evoked responses originate from a distributed network of sources) and averaged across hemispheres) was fitted to the whole time-window of auditory stimulation (0–3000 ms). In this model, the strengths of both intrinsic and extrinsic connections were allowed to be independently modulated by two experimental factors, regularity and alphabet size. This full model was inverted using a variational Bayes scheme to obtain the free-energy approximation to its log-evidence (Friston et al., 2007). The parameters of the full model were optimized using Bayesian model reduction (Friston et al., 2016), which calculates the posterior estimates of all reduced models (each allowing for different combinations of connections to be modulated by regularity and alphabet size). The posterior estimates of all reduced models were averaged using Bayesian model averaging (Penny et al., 2010) and treated as priors for subsequent Bayesian belief updating analysis.

To estimate how the intrinsic connectivity parameters changes over time, and to what extent these changes are explained by the emergence of sequence predictability, we inverted the two-source DCM using cross-spectral densities of consecutive time windows (width 500 ms, time-step 100 ms; smoothed over time with a 300 ms full-width at half-maximum Gaussian kernel). Bayesian belief updating was performed by treating the posterior parameter estimates from the previous time window as priors for the subsequent time window (cf. Cooray et al., 2015; Papadopoulou et al., 2015). We considered five alternative models, where regularity and alphabet size could modulate one of five sets of connections: extrinsic connections only (similar to the effects of expectation by Auksztulewicz and Friston, 2015); the intrinsic gain of inhibitory interneurons, with or without extrinsic modulation (similar to the effects of attention in Auksztulewicz and Friston, 2015); and the intrinsic gain of superficial pyramidal cells, with (Moran et al., 2013) or without (Feldman and Friston, 2010) extrinsic modulation. The parameters not updated in a given model were treated as constants (using the averaged posterior parameter estimates from the whole time-window).

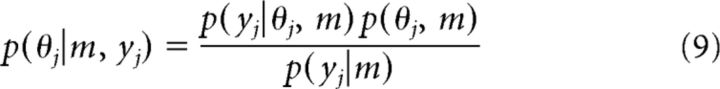


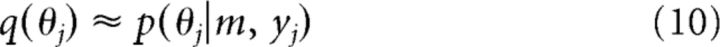


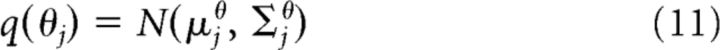


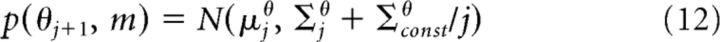
 The above equations describe the Bayesian belief updating approach, where the posterior *q*(θ*_j_*) approximates the conditional parameter density *p*(θ*_j_*|*m*, *y_j_*) given data *y* and model *m*. Under the Laplace approximation, both the prior parameter density *p*(θ*_j_*, *m*) and the variational density assume a Gaussian form *N*(μ_*j*_^θ^, Σ_*j*_^θ^). The conditional mode μ_*j*_^θ^ and covariance Σ_*j*_^θ^ from a *j*th time window define the prior density for the subsequent time window *j*+*1*. To avoid the shrinking of covariance, at each time step a constant term Σ_*const*_^θ^ is added to the updated prior covariance, divided by the time window index *j*.

The time-resolved parameter estimates of the modulatory effects of intrinsic connections were treated as predictors in an elastic-net-penalized multi-response linear regression (Simon et al., 2013) of the sensor-level main and interaction effects of regularity and alphabet size (cf. Barascud et al., 2016). To avoid circularity in using source-level data to predict sensor-level data, we recalculated the sensor-level effect time courses after low-pass filtering the data at 8 Hz (given that the DCM parameter estimates were based on source-level 8–128 Hz activity). To verify that the lower- (<8 Hz) and higher-frequency (8–128 Hz) data components are indeed independent in our dataset, we correlated the time course of the grand-mean RMS during acoustic stimulation (averaged across conditions) with the time course of the first principal component (PC) of induced 8–128 Hz power (averaged across conditions; first PC explaining 99.11% of the spectral variance over stimulation time). The ensuing correlation was not significant (*r* = −0.136, *p* = 0.341). Thus, mean RMS (<8 Hz) did not correlate with mean induced power (8–128 Hz). Similarly, the SD of the RMS across conditions did not correlate with the first PC of the SD of induced power across conditions (PC: 92.78% of spectral variance; *r* = 0.0133, *p* = 0.352), suggesting that lower- and higher-frequency differences between experimental conditions explained largely non-overlapping variance components. The eight predictors (2 experimental factors × 4 modulatory parameters) were up-sampled to match the sampling rate of the sensor-level data (600 Hz) using MATLAB's resample function with nearest-neighbor interpolation, and smoothed with a Gaussian kernel (SD 150 ms). In the regression, we set the elastic-net mixing parameter α to 0.95 (yielding a relatively sparse predictor structure). This analysis returned an ordering of the eight DCM parameters according to how well they explain sensor-level effects.

## Results

The effects of the two orthogonal manipulations of predictability (regularity and alphabet size) on the sustained MEG field were previously reported by Barascud et al. (2016) and are presented in Figure 2. Both regularity and alphabet size contribute to the sustained field amplitude during acoustic stimulation. Note that the dynamics of the sustained response in Figure 2 differ slightly from those in Barascud et al. (2016) due to the different preprocessing of the data (e.g., presence of a high-pass filter in the present study and no principle component analysis), as well as the different selection of sensors over which to measure global responses. The early (<1 s) main effect of regularity (REG > RAND), as well as the interaction effect of regularity and alphabet size (defined as REG5 > REG15 × RAND5 < RAND15) can be explained by the statistics of the stimuli: sequences can only be categorized as regular once a certain number of repetitions has occurred. Barascud et al. (2016) demonstrate that an ideal observer requires a cycle plus four tones to distinguish regular from random sequences and that human listener performance closely matches this threshold. Therefore, the early effects observed here likely arise because different REG conditions take a different time to be “discovered”. The significant effect further along in the sequence (>2.2 s; see Fig. 2*B*) occurs well after all the REG patterns have been discovered and reflects the observed increased sustained field amplitude to ongoing regularity, relative to random sequences. In the following, we analyze the induced neural responses observed in these data, testing for their modulation by regularity and alphabet size. We then use dynamic causal modeling of cross-spectral density to explain the effects of predictability on induced responses in terms of synaptic and connectivity parameters (that mediate gain modulation). Finally, we use the model-based trajectories of synaptic gain parameters to explain the sensor-level sustained field amplitude modulations, linking the slowly evolving changes in synaptic efficacy to effects observed at the MEG sensor level.

### Evoked responses originate from a distributed network of sources

**Figure 3.**
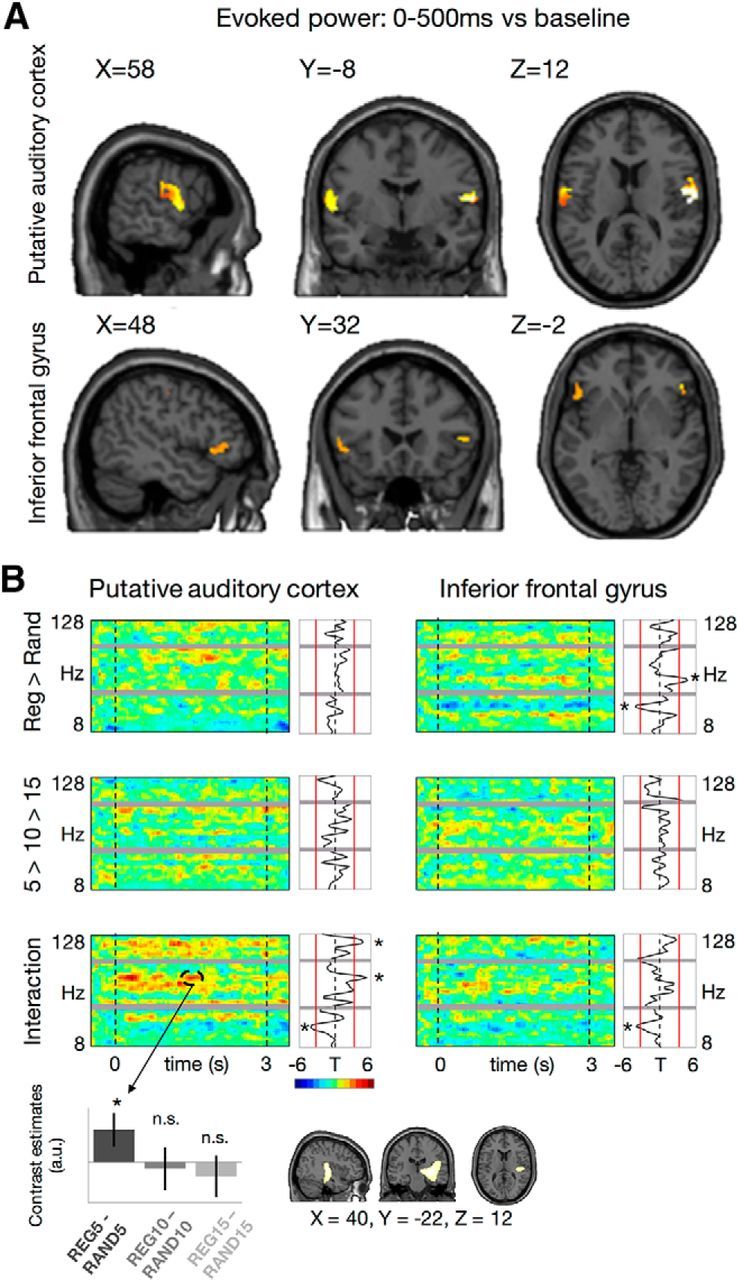
***A***, Source reconstruction. Sources of strongest activity evoked by the onset of the auditory sequences were localized to bilateral putative auditory and inferior frontal cortices. ***B***, Source-level time-frequency contrasts. Each panel shows *t* statistics as time-frequency maps (large panels; highlighted cluster exceeds corrected significance threshold at FWE = 0.05) and time-averaged frequency spectra (small panels; red lines: thresholds of significance at FWE = 0.05). Gray bars indicate notch filter and harmonic. Bar plot inset shows *post hoc t* tests and 95% confidence intervals for the significant time-frequency cluster showing an interaction of regularity and alphabet size on induced power in auditory cortex (82 Hz, 1500 ms), dominated by the difference in high-frequency power between REG5 and RAND5 conditions. Source activity inset shows that the high-frequency interaction effect at 1500 ms can be specifically assigned to early auditory regions in a whole-brain analysis. Asterisks mark significance after FWE correction.

The early sensor-level response evoked by auditory stimulus onset (0–500 ms, relative to baseline) was localized to bilateral cortical sources in two regions (Fig. 3*A*): the putative auditory cortex (AC), including the transverse temporal gyrus and planum temporale [MNI coordinates left: (−60, −4, 14), right: (58, −8, 12)], and the inferior frontal gyrus [IFG; MNI left: (−48, 22, −2); right: (48, 32, −2)]. Although the auditory cortex was not indicated as the most probable label (Neuromorphometrics: central operculum, left: 21.2–40.3%, right: 42.5–55.1%; Talairach: postcentral gyrus, left: 73.4%, right: 63.6%), the auditory regions were also included in probabilistic labeling in both atlases (Neuromorphometrics: right planum temporale 18.4%, right transverse temporal gyrus 11.3%, left transverse temporal gyrus 4.8%, left planum polare 10.9%, left superior temporal gyrus 5.4%; Talairach: left transverse temporal gyrus 17.2%, right transverse temporal gyrus 4.2%). Given our strong a priori hypothesis that acoustic stimuli at early latencies would be processed in auditory regions, the two clusters will thus be referred to as (putative) auditory cortex. The sources generating the evoked response match well those identified by Barascud et al. (2016) as more activated for REG than for RAND, suggesting that the process of regularity detection involves an increase in activity in sources that respond to the unfolding sequences. Because no differences in time-frequency responses were observed between homologous areas in different hemispheres (two *F* tests: left vs right AC and left vs right IFG; all voxels *p*_FWE_>0.05), for subsequent analysis of time-frequency effects induced by our experimental manipulations, homologous areas were averaged across hemispheres.

### Regularity and alphabet size modulates high-frequency-induced responses

Regularity and alphabet size had an interactive effect (with the interaction formally defined as REG5 > REG15 × RAND5 < RAND15, but see contrast estimates in Fig. 3*b*) on induced high-frequency power (82 Hz) in AC (Fig. 3*B*). This interaction was seen during ongoing stimulation (1500 ms; peak-level *t* value 4.50; cluster-level, FWE-corrected *p* = 0.028). *Post hoc* tests revealed that this interaction effect was due to an increase in power for the REG5 condition versus RAND5 condition but not for the other alphabet sizes (paired *t* tests: REG5 vs RAND5, *p* = 0.001; REG10 vs RAND10, *p* = 0.528; REG15 vs RAND15, *p* = 0.173; Fig. 3*B*, see inset with contrast estimates).

To establish that the interaction can be assigned to high-frequency activity in early auditory regions, as opposed to reflecting signal leakage from other sources, we performed an additional control analysis. Here, we estimated the whole-brain sources of high-frequency activity for each experimental condition using dynamic imaging of sources. The effect at 1500 ms was evident as an interaction between regularity and alphabet size on high-frequency source power within a cluster encompassing the right putative auditory cortex, including the transverse temporal gyrus and planum temporale [Fig. 3*B*; MNI: (40, −22, 12); peak-level *t* value 3.65; cluster-level, FWE-corrected *p* = 0.037] and extending into the right thalamus [MNI: (10, −20, 0); peak-level *t* value 3.88]. The medial part of the cluster is likely due to a beamforming bias toward the center of the head (Barnes and Hillebrand, 2003), and similar results have been previously obtained in localization of gamma-band activity induced by auditory stimulation (Sedley et al., 2012). Using a more liberal threshold (*p* < 0.05, uncorrected) revealed an additional left-lateralized source in the superior temporal gyrus [peak MNI: (−52, −50, 18); data not shown]. Together, this analysis confirmed that the interaction could be attributed to high-frequency responses in early auditory sources.

Thresholding the statistical parametric maps of induced responses at a more liberal threshold (*p* < 0.005 peak-level; cluster-corrected at *p*_FWE_ < 0.05) revealed additional clusters of effects (see Table 1 for exact frequencies, latencies, and statistics): increased high-frequency (86–88 Hz) and decreased alpha-band (12–14 Hz) activity for REG versus RAND sequences in the AC; decreased beta-band (36–38 Hz) activity for REG versus RAND sequences in the IFG; as well as an interaction in high-frequency (116–120 Hz) power in the AC, and in the low-frequency (26–30 Hz) power in the IFG.

**Table 1.** Effects of regularity on induced power (time-frequency domain)

Effect	Region	Peak frequencies, Hz	Peak latencies, ms	*t*_(1,12)_ peak-level	*p* (cluster-level FWE)
Regularity (REG > RAND)	AC	86	1800	4.37	0.003
		88	2000	3.14	
Regularity (RAND > REG)	AC	12	2200	4.03	<0.001
		12	2050	3.87	
		14	2350	3.40	
	IFG	38	2750	3.73	0.016
		36	2450	2.75	
		38	1100	3.65	0.005
		36	1300	3.07	
Interaction (REG5 > REG15 × RAND5 < RAND15)	AC	120	1250	3.86	0.006
		118	1150	3.76	
		116	1450	3.23	
Interaction (REG5 < REG15 × RAND5 > RAND15)	IFG	26	1050	3.70	0.007
		30	950	3.15	

Statistical parametric maps (over frequency) thresholded at *p* < 0.005 and corrected at FWE = 0.05.

Pooling across time (Fig. 3*B*, see small panels), stimulus regularity significantly increased the power of high gamma (64 Hz) activity and decreased power in a lower frequency band (36 Hz) in IFG. Furthermore, regularity and alphabet size had interactive effects on induced responses in both AC and IFG. In AC, high-frequency power (with 2 peaks at 82 and 120 Hz) was stronger for REG5 than REG15 sequences, as opposed to less pronounced differences between the RAND conditions. The opposite interactive effect (REG5 < REG15) was found in the beta band (30 Hz) in both regions (Fig. 3*B*; Table 2).

**Table 2.** Effects of regularity on induced power (time-averaged spectra)

Effect	Region	Peak frequencies, Hz	*t*_(1,12)_ peak-level	*p* (cluster-level FWE)
Regularity (REG > RAND)	IFG	64	4.60	0.005
Regularity (RAND > REG)	IFG	36	4.00	0.005
Interaction (REG5 > REG15 × RAND5 < RAND15)	AC	120	4.70	<0.001
		82	5.32	<0.001
Interaction (REG5 < REG15 × RAND5 > RAND15)	AC	30	4.09	<0.001
	IFG	30	3.92	0.005

Statistical parametric maps (over frequency) thresholded and corrected at FWE = 0.05.

### Intrinsic connectivity modulation tracks stimulus regularity over time

To model the spectral components observed in our data, a DCM comprising two cortical sources (AC and IFG) and 2 experimental factors, regularity and alphabet size, which modulated the gain parameters in both sources, was fitted to the cross-spectral density estimated over the whole 0–3000 ms stimulation time window. The posterior parameter estimates of this whole-window model are shown in Table 3. The posterior parameter estimates of this model were used as priors for a subsequent sliding time-window analysis based on Bayesian belief updating (see Materials and Methods). This Bayesian belief updating effectively models the cumulative effects of stimulus regularity and alphabet size on intrinsic and/or extrinsic connectivity.

**Table 3.** Optimized DCM connectivity parameters

Parameter name	Prior mean	Prior (log) variance	Posterior mean	Posterior variance	Description
A{1}(2,1)	0	1/16	−1.0919	0.0283	Ascending connection from AC to IF (SP to SS)
A{2}(2,1)	0	1/16	−0.0901	0.0553	Ascending connection from AC to IF (SP to DP)
A{3}(1,2)	0	1/16	−0.0877	0.0341	Descending connection from IF to AC (DP to SP)
A{4}(1,2)	0	1/16	−0.3759	0.0527	Descending connection from IF to AC (DP to II)
G(1,:)	−1.39; −2.02	1/8; 1/8	−1.3455; 0.2480	0.0568; 0.0157	Intrinsic connections in AC (SP gain; II gain)
G(2,:)	−1.39; −2.02	1/8; 1/8	−1.1852; −2.2009	0.0498; 0.0575	Intrinsic connections in IF (SP gain; II gain)
B{1}(1,1)	0	1/8	−0.1324	0.0013	Modulation of SP gain in AC by regularity
B{1}(2,2)	0	1/8	0.0624	0.0001	Modulation of SP gain in IF by regularity
B{2}(1,1)	0	1/8	0.0001	0	Modulation of SP gain in AC by alphabet size
B{2}(2,2)	0	1/8	−0.0437	0.0001	Modulation of SP gain in IF by alphabet size

The posterior means were used as priors while inverting the initial time window in the Bayesian belief updating analysis.

**Figure 4.**
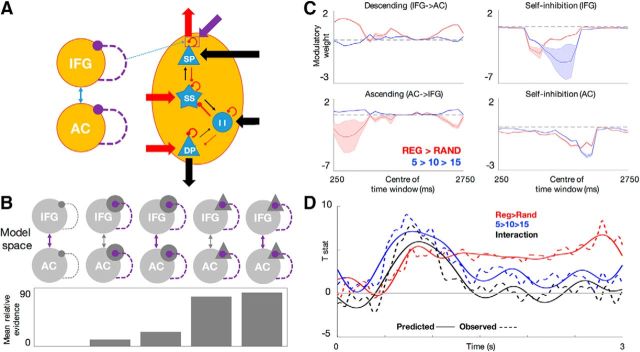
***A***, Basic model architecture of a two-source DCM (left) and a canonical microcircuit used to model the responses of each source (right), comprising four neuronal populations (SP, DP, SS, II) characterized by excitatory (black) and inhibitory (red) connections between populations (thin arrows), gain parameters (self-inhibitory connections), as well as sending and receiving connections to and from different regions (thick arrows; arrow width represents prior connection strength). Modulatory connections (here, superficial pyramidal gain) are shown in purple. ***B***, Five alternative models of connections modulated by alphabet size and regularity (extrinsic connections only; inhibitory interneuron gain; extrinsic connections and inhibitory interneuron gain; superficial pyramidal gain; extrinsic connections and superficial pyramidal gain) were compared against one another. Bottom, Log-model evidence (averaged across time windows), relative to the weakest model. ***C***, Time courses of modulatory parameters. Red indicates modulation by sequence regularity (relative to a RAND baseline); blue indicates modulation by alphabet size (relative to an R5 baseline); shaded areas indicate 95% confidence intervals. The predictive effects of alphabet size in RAND are described by the modulatory effects of alphabet size, relative to a RAND5 baseline. However, the predictive effects of alphabet size in REG are due to cumulative effects of both REG > RAND and alphabet size. ***D***, DCM parameters as predictors of the sensor-level effects. Sensor-level effects of regularity and alphabet size as observed (dashed lines) and predicted by the DCM parameters (solid lines). Please note that the observed sensor-level effects correspond to those shown in Figure 2*B*, after low-pass (<8 Hz) filtering the data.

First, we compared five alternative models, in which different subsets of connectivity parameters could be modulated by the experimental factors (Fig. 4*B*). By accumulating model evidence across time windows, we identified the model with changes in both intrinsic connectivity (gain of superficial pyramidal cells within regions) and extrinsic connectivity (ascending and descending connections between regions) as the best model. Note that the intrinsic gain parameters are modeled as recurrent collaterals mediating the self-inhibition of superficial pyramidal cells (i.e., each excitatory population is equipped with its own inhibitory population to model gain control): weaker self-inhibition (stronger disinhibition) will result in greater (or faster) postsynaptic responses to intrinsic inputs from other populations within a given region as well as extrinsic afferents from other regions. The difference between the pooled log-model evidence of the winning and the second-best model (in which regularity and alphabet size could only modulate intrinsic connectivity) was 7.04 nats, corresponding to very strong evidence in favor of the winning model (Penny et al., 2004).

Following model inversion and selection, we investigated the time-resolved changes in extrinsic and intrinsic connectivity mediated by the two experimental factors (Fig. 4*C*). Shortly after sequence onset, both ascending and descending connections were modulated by regularity and alphabet size. Regular sequences (and sequences with larger alphabet sizes) induced weaker excitatory ascending connectivity and stronger inhibitory descending connectivity, relative to random sequences (and sequences with smaller alphabet sizes). After this initial effect on extrinsic connectivity, the intrinsic gain in IFG was increased (due to pyramidal disinhibition) first by regularity (peaking at ∼1050 ms post-sequence onset) and then by alphabet size (peaking at ∼1550 ms), with smaller alphabet sizes associated with stronger gain increase. Finally, both regularity and alphabet size increased neuronal gain in AC later in the sequence (peaking at 1850 and 1950 ms, respectively). Note that we did not have to model the interaction between regularity and alphabet size explicitly. This is because the distributed and nonlinear generation of spectral responses in DCM renders the main effects on intrinsic and extrinsic connectivity a sufficient account of the data.

### Model-based parameters explain sensor-level sustained field shifts

To link these changes in connectivity (i.e., synaptic efficacy) to observed sensor-level effects, we entered the eight modulatory parameter time series into an elastic-net-penalized multi-response linear regression (Simon et al., 2013), in which the effects of regularity and alphabet size on sensor-level sustained field amplitude were predicted by the DCM parameters. A regression model with the minimum residual minimum-square error (MSE) indicated that the eight DCM parameters explained 82.99% MSE (Fig. 4*D*). The first parameter included in the model (according to the ratio of MSE explained) corresponded to the intrinsic gain modulation in the IFG by alphabet size, followed by gain modulation in AC by regularity. All eight DCM parameters were significant predictors of sensor-level effects in the most parsimonious model accounting for <1 SE of minimum MSE. This is a nontrivial result because the DCM estimates of synaptic efficacy were based upon (cross-spectral) data from which sustained (<8 Hz) responses were removed.

In summary, the cumulative effects of different sorts of predictability on (extrinsic and intrinsic) synaptic connectivity furnish a sufficient (and accurate) prediction of the remarkable changes in sustained field amplitude described by Barascud et al. (2016), in a way that is entirely consistent with generalized (Bayesian filtering or) predictive coding.

## Discussion

Given recent evidence that regularly repeating tone sequences produce sustained MEG fields at higher amplitudes than random sequences (Barascud et al., 2016; see also Southwell et al., 2017 for a replication in EEG, and Sohoglu and Chait, 2016 who demonstrated the same effect for a different stimulus), we aimed to provide a mechanistic explanation of the effects of stimulus predictability on sustained responses. We identified significant main effects of sequence regularity, and their interaction with alphabet size, on the amplitude of induced gamma activity in the auditory and frontal cortices. In the auditory cortex, sequences with fewer tones (smaller alphabet size) induced stronger gamma activity, relative to sequences with more tones; especially when these sequences were regular (Fig. 3*B*). This interactive effect of regularity and alphabet size has been observed half way through the sequence (∼1500 ms), as well when pooling across time (from 750 post-onset until the end of the stimulus), and could be explained by a significant difference between REG5 and RAND5 conditions.

A further inspection of the effects of experimental manipulations on induced activity in the auditory cortex revealed an increase of high-frequency (86–88 Hz) activity at the expense of alpha-band (12–14 Hz) activity induced by regular sequences, as opposed to random sequences (Table 1). Regular sequences also induced a shift toward higher frequency activity in the IFG. The effects on induced gamma (64 Hz) there overlapped with the frequency range in which sustained auditory responses to pitch stimuli have been previously reported (Griffiths et al., 2010). The shift from lower to higher frequencies under predictable stimulation conditions is consistent with previous reports of gamma-band synchronization and alpha-band desynchronization due to contextual predictability (Arnal et al., 2011; Todorovic et al., 2011; Bauer et al., 2014; Brodski et al., 2015; Sedley et al., 2016). Strong induced high-frequency responses are consistent with generalized predictive coding, under which stimulus predictability is thought to increase postsynaptic gain (Chawla et al., 1999; Bastos et al., 2012; Auksztulewicz and Friston, 2016). Generalized predictive coding refers to the generalization of Bayesian filtering schemes to estimate both latent or hidden states generating data and the parameters of the generative model; i.e., generalization to both inference and learning. Crucially, these parameters include the precision of random effects. In summary, generalized predictive coding suggests that the brain predicts both the content of sensory input and (on a longer time scale) the predictability of that input (Kanai et al., 2015). Predictions of predictability determine the precision of sensory prediction errors, thought to be encoded by the postsynaptic gain of superficial (pyramidal) cells reporting prediction errors. Increases in predictability will therefore increase synaptic rate constants (i.e., increase intrinsic connectivity or synaptic gain), which necessarily increases the frequency of neuronal activity (i.e., decrease synaptic time constants; cf. Chawla et al., 1999; Pinotsis et al., 2013). The resulting gain modulation may mediate attentional effects (Brown and Friston, 2012; Auksztulewicz and Friston, 2015; but see Southwell et al., 2017) and underlie phenomena such as communication through coherence (Fries, 2005).

Dynamic causal modeling of cross-spectral density revealed that changes in intrinsic connectivity showed the strongest modulation by both manipulations of predictability. Increased synaptic gain in the IFG due to sequence regularity was followed by additional frontal gain increase by small alphabet size. Later in the sequence, both factors had a disinhibitory effect in the auditory cortex. These results suggest that even under passive stimulation, after an initial period of evidence accumulation, the estimated precision of sensory inputs is propagated from higher (IFG) to lower (AC) regions and dynamically modulates sensory gain. Although both regions have been implicated in perceptual inference, predominantly in mismatch negativity paradigms (Opitz et al., 2002; Molholm et al., 2005; Garrido et al., 2009; Phillips et al., 2015), our study is the first to show the cumulative influences of stimulus predictability on gain modulation along the auditory processing hierarchy. The latency difference between disinhibitory effects on frontal and sensory cortex speaks to the role of the IFG as contextualizing processing within sensory cortical regions (Phillips et al., 2015). Furthermore, consistent with the modulation of intrinsic frontal gain by alphabet size, the IFG was previously found to reflect quantitative magnitude in its oscillatory activity (Spitzer et al., 2014). Our study accommodates these previous findings and suggests a broader role of the IFG as originating descending signals about estimated precision or predictability of the sensory environment. More generally, our results illustrate the dynamic and hierarchical deployment of postsynaptic gain modulation by contextual predictability, in line with its role in optimizing the precision of ascending prediction errors in predictive coding (Friston, 2005; Auksztulewicz and Friston, 2016). For instance, the gradual transition from augmentative to suppressive effects of predictions due to learning (Müller et al., 2013) has been hypothesized to reflect early gain modulation (increased precision) in hierarchically higher regions, gradually deployed in hierarchically lower (e.g., sensory) regions at later stages (Auksztulewicz and Friston, 2016), similar, albeit at a longer time scale, to the effects observed here.

In addition to intrinsic gain modulation, predictability also modulated extrinsic connectivity between the AC and the IFG. Regular sequences were associated with weaker excitatory ascending connectivity and stronger inhibitory descending connectivity than random sequences, a pattern of results well explained by a relative increase in the precision of descending predictions, when processing predictable stimulus sequences (Garrido et al., 2009). This effect suggests that forward and backward message passing is sensitive to predictability at early latencies. A similar (although markedly less pronounced) inhibition of ascending drive was seen for sequences with large alphabet sizes compared to small alphabet sizes, consistent with previous reports that frequency-specific adaptation is modulated by spectral variance of acoustic stimulation (Herrmann et al., 2013). Nevertheless, in DCM, the only distinction between extrinsic and intrinsic connectivity is the distinction between the gains of postsynaptic responses to extrinsic afferents from other sources, as opposed to intrinsic afferents from neuronal populations within the same source (in our case, recurrent collaterals mediating self-inhibition of superficial pyramidal cells).

To establish whether modulations of (extrinsic and intrinsic) synaptic gain can explain the observed sensor-level sustained fields, cumulative changes in DCM parameter estimates were treated as (multilinear) predictors of sustained field changes at the sensor level. The parameters based on fast (8–128 Hz) activity explained 82.99% of the variance of slow sustained (RMS <8 Hz) effects (Fig. 4*D*). Although extrinsic as well as intrinsic connectivity parameters contributed to explaining source-level (Fig. 4*B*) and sensor-level data (Fig. 4*D*), the two parameters with the greatest contribution to explaining RMS effects corresponded to intrinsic gain modulation (in the IFG by alphabet size, and in AC by sequence regularity). This finding corroborates the interpretation of sustained field shifts as resulting from cumulative increases in synaptic efficacy or gain during exposure to predictable sensory streams (Barascud et al., 2016) and provides a direct, biophysically grounded link between induced synchronous gain and sustained responses. Furthermore, this link is consistent with (generalized) predictive coding accounts of perceptual inference that entails not only the prediction of stimuli but also a prediction of their predictability.
